# Eight weeks of creatine monohydrate supplementation is associated with improvements in muscle size in Alzheimer's disease

**DOI:** 10.1002/alz70860_105967

**Published:** 2025-12-23

**Authors:** Aaron N Smith, Debra K Sullivan, Trent J Herda, Matthew K Taylor

**Affiliations:** ^1^ University of Kansas Medical Center, Kansas City, KS, USA; ^2^ University of Kansas, Lawrence, KS, USA

## Abstract

**Background:**

Alzheimer's disease (AD) directly reduces skeletal muscle mass—thereby impairing physical function and potentially accelerating disease progression—and preclinical evidence suggests that skeletal muscle may also influence AD risk and pathology via the muscle‐brain axis. Consequently, interventions to increase muscle size could help prevent the functional decline associated with AD. Although creatine monohydrate (CrM) is widely recognized to increase muscle mass in various populations, its benefits have yet to be examined in AD. This single‐arm pilot study investigated whether eight‐week CrM supplementation period impacts leg muscle cross‐sectional area (mCSA) in older adults with AD.

**Method:**

Data from 18 participants with probable AD who completed a one‐arm, eight‐week trial investigating the feasibility and preliminary efficacy of 20 g/day of CrM in AD were analyzed. At baseline and eight weeks, ultrasonography imaging was completed on the rectus femoris (RF) and vastus medialis (VM). mCSA was calculated in cm^2^ using ImageJ software (National Institutes of Health). We conducted paired t‐tests to test for 8‐week change in mCSA in the RF and VM. Statistical significance was set at *p* < 0.05.

**Result:**

Participants were 65% male and had a mean age of 73.1 ± 6.3 years at baseline. The mean mCSA for the rectus femoris (RF) and vastus medialis (VM) at baseline were 7.6 ± 2.5 cm^2^ and 10.1 ± 3.0 cm^2^, respectively. After 8 weeks of CrM supplementation, the mean mCSA increased to 7.8 ± 2.7 cm^2^ for the RF (*p* = 0.03) and 10.2 ± 3.1 cm^2^ for the VM (*p* = 0.01). BMI did not change.

**Conclusion:**

This pilot study provides the first evidence that supplementing 20 g/day of CrM for eight weeks may increase muscle size in individuals with AD. Although these gains were modest, they could still hold clinical significance given that AD is a progressive disease typically marked by muscle loss. Thus, CrM supplementation may offer a strategy to maintain muscle mass or slow functional decline. Future randomized controlled trials with extended durations investigating neuromuscular outcomes are warranted to confirm and expand upon these findings.

